# Gallbladder Radiation Protection in SIRT—Quantitative Anatomical Study of Hepatic Vasculature

**DOI:** 10.3390/jcm8101531

**Published:** 2019-09-24

**Authors:** Piotr Piasecki, Krzysztof Brzozowski, Piotr Ziecina, Marek Wierzbicki, Anna Budzynska, Andrzej Mazurek, Miroslaw Dziuk, Maciej Maciak, Edward Iller, Jerzy Narloch

**Affiliations:** 1 Department of Interventional Radiology, Military Institute of Medicine, Szaserów 128, 04-141 Warsaw, Poland; piotr_piasecki@wp.pl (P.P.); kbrzozowski@wim.mil.pl (K.B.); pziecina@wim.mil.pl (P.Z.); mwierzbicki@wim.mil.pl (M.W.); 2 Department of Nuclear Medicine, Military Institute of Medicine, Szaserów 128, 04-141 Warsaw, Poland; abudzynska@wim.mil.pl (A.B.); andrzej_mazurek@wim.mil.pl (A.M.); mdziuk@wim.mil.pl (M.D.); 3 National Centre for Nuclear Research, Andrzej Soltan 7, 05-400 Otwock, Poland; Maciej.Maciak@ncbj.gov.pl; 4 National Centre for Nuclear Research, Radioisotope Centre POLATOM, Andrzej Soltan 7, 05-400 Otwock, Poland; Edward.Iller@polatom.pl

**Keywords:** SIRT, gallbladder radiation, vascular anatomy, hepatic artery, cystic artery, extrahepatic leaking

## Abstract

**Introduction:** This study was designed to assess quantitatively a safe position of the microcatheter during the SIRT (Selective Internal Radiation Therapy) procedure, in order to minimize the risk of non-target spheres leaking. **Materials and Methods:** Retrospective analysis of the distance of the tip of the microcatheter from coiled or patent non-target arteries was measured during ^99m^Tc-MAA work-up procedure. Frequency of extrahepatic leaking during work-up and SIRT procedures was evaluated. **Results:** There were 85 patients who underwent 98 work-up procedures. There were 64 radioembolizations. There were 44 gastroduodenal, 51 right gastric, and 54 cystic artery embolizations performed. Extrahepatic ^99m^Tc-MAA leaking was observed in 33 cases: 16 to gallbladder, four to a gastric wall, nine to the duodenum, one to the intestinal wall, and three to the abdominal wall. Leak in ^99m^Tc-MAA was also related to the presence of additional arteries (*p* = 0.009). There were 34 proximal and 31 distal to cystic artery ^99m^Tc-MAA injections resulting in 12 vs. four leaks, respectively (*p* = 0.039, RR-2.5). Mean distance of the tip of the microcatheter from the origin of the cystic artery was 20 mm (minimum of 2.1 mm and maximum of 53 mm) proximally and 10 mm (minimum 1 mm and maximum 51 mm) distally (ns). **Conclusions:** Leaking in ^99m^Tc-MAA (^99m^Tc - labelled macroaggregated albumin) was related to the presence of additional arteries. Regardless of cystic artery embolization, it is 2.5 times safer to inject microspheres distal to its origin, compared to proximal injection. Cystic artery origin relative to the right hepatic artery division usually necessitates embolization of the former.

## 1. Introduction

Safety and efficiency of SIRT (Selective Internal Radiation Therapy) procedures depend on proper intrahepatic deposition of millions of ^90^Y-micorspheres. There are plenty of methods described in the guidelines and literature to achieve these goals during work-up and treatment procedures [1,2,3,4,5,6,7,8]. The key point of both procedures is to properly position the tip of the microcatheter within hepatic arteries. It may guarantee an equal spread of microspheres within the liver tissue without its extrahepatic deposition. 

According to the guidelines, the tip of the microcatheter should be placed “in a safe position” in the hepatic artery, i.e., in a proper hepatic artery for non-selected spread of radiated microspheres into two liver lobes or, for more selective injection, in the right or left hepatic artery. This is often challenged by many anatomical variants of liver blood supply, with a need to coil at least a few non-target arteries. Cystic artery (CA), right gastric artery (RGA), and sometimes gastroduodenal artery (GDA) are most often targeted to secure gastrointestinal organs against radiation in case of blood reflux during the SIRT procedure. Other arteries arising from hepatic arteries are also observed (i.e., supra-duodenal artery, falciform artery, and additional cystic or right gastric arteries). Sometimes, it is not possible to coil these arteries due to their small lumen or difficult tortuous anatomy. In these cases, the risk of adverse events during the SIRT procedure related to suboptimal microcatheter position is increasing dramatically. There is an ongoing discussion in the literature, whether there is a need to embolize CA, RGA, or GDA with new evidence emerging in support of leaving them patent. When there is evidence on recanalization of previously coiled arteries [2,9,10,11].

There is no sufficient recommendation regarding what constitutes “the safe microcatheter position” and there is still space for a strictly personal interpretation among interventional radiologists, dependent on skills, expertise, and willingness to risk. 

The aim of this study was to assess quantitatively a safe position of the microcatheter during the SIRT procedure, in order to minimize the risk of non-target spheres leaking.

## 2. Materials and Methods

The study was approved by Local Ethical Committee—decision number: 24/WIM/2009. Informed consent was obtained before work-up and SIRT procedures.

Retrospective analysis of angiography data from preparatory and SIRT procedures was performed in consecutive patients planned for treatment between June 2009 and September 2018. Both work-up and ^90^Y-radioembolisation procedures were conducted according to the guidelines [7,8,12,13,14]. The safe position for the tip of the microcatheter was chosen at the discretion of the interventional radiologist to avoid a potential extrahepatic leak and ensure uniform distribution of microspheres, as per definition. Administration of 120–180 MBq of ^99m^Tc-MAA (^99m^Tc-labelled macroaggregated albumin) into hepatic arteries (proper hepatic artery or separately into right or left hepatic artery) was performed in each work-up procedure. 

In the ^90^Y-radioembolisation procedure, resin microspheres were injected from the same microcatheter position and ^90^Y-bremmstrahlung SPECT/CT was performed. One session of resin microspheres (Sirtex, Australia) treatment was used for the study. The distance of the tip of the microcatheter from coiled or patent non-target arteries was measured during the ^99m^Tc-MAA work-up procedure. All potential sites of the extrahepatic leak were recorded.

In the next step, frequency and site of the actual extrahepatic leak during work-up and SIRT procedures was evaluated (in ^99m^Tc-MAA and ^90^Y-SPECT/CT, respectively). The following non-target arteries were considered: cystic artery (CA), right gastric artery (RGA), gastroduodenal artery (GDA), falciform artery (FA), supra-duodenal artery (SA), and additional cystic or right gastric arteries (aCA and aRGA respectively). In selected cases, dyna-CT had to be performed in order to confirm their presence. 

**Figure 1:**
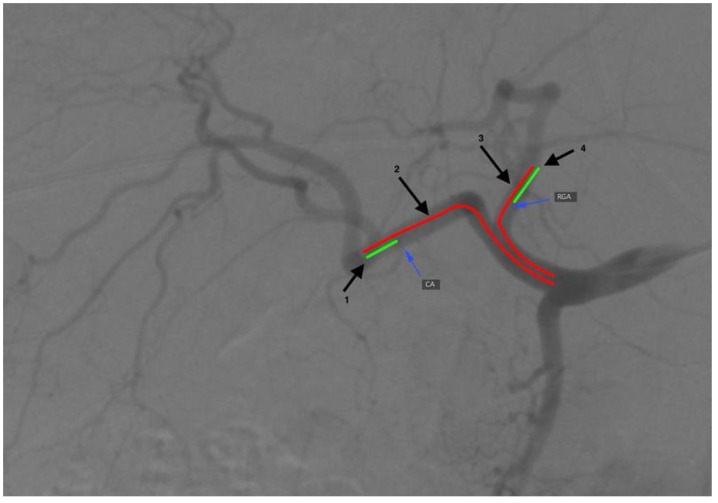
A general scheme presenting the measurement procedure. Thin arrows indicate origins of the cystic artery (CA) and the right gastric artery (RGA). Numbers at thick arrows (1–4) indicate distances (lines) of the tip of the microcatheter from the cystic artery, the gastroduodenal artery (for right lobe injection), the gastroduodenal artery (for left lobe injection), and the right gastric artery, consecutively.

The Osirix (Pixmeo SARL, Geneva, Switzerland) software was used to carry out measurements on DSA (digital subtraction angiography) images (Figure 1). Three-month follow-up was used to observe potential early adverse events linked with extrahepatic radiated microspheres spread, (i.e., cholecystitis, gastric ulcer, or skin radiation). It consisted of a patient’s physical examination, liver function tests, and complete blood count assessments at 1–5 days and every 2–3 weeks as well as a follow-up CT (computed tomography) two to three months after the SIRT procedure. The presence of adverse events linked with extrahepatic arteries embolization in the work-up procedure was assessed with Common Terminology Criteria for Adverse Events (CTCAE v 3.0). 

## 3. Results

Between June 2009 and September 2018, 85 patients (57 men, 28 women) had undergone 98 work-up procedures. Overall, there were 64 ^90^Y-radioembolisations performed. The remaining 21 patients were not given SIRT due to: worsening of health status, iatrogenic hepatic artery dissection, development of extrahepatic collaterals that could not be embolized, a persistent leak with no identifiable vessel, disadvantageous anatomy, or retracted consent. Mean time between the work-up and SIRT procedures was 15 days.

**Figure 2:**
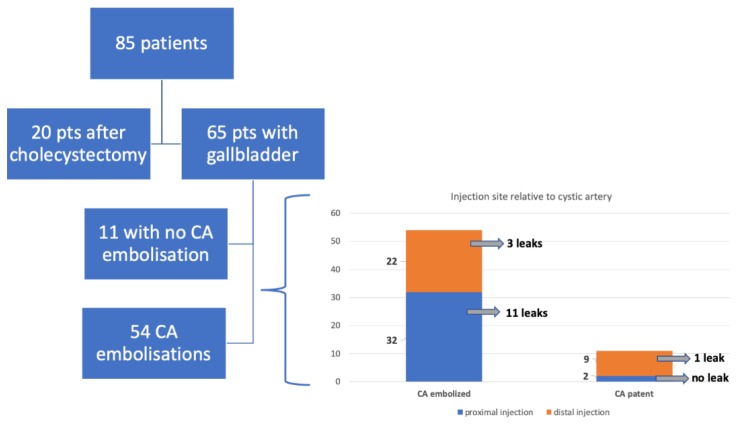
Flowchart presenting the sites of extrahepatic leaks.

There were 44 GDA, 51 RGA, and 54 CA embolizations performed. Extrahepatic ^99m^Tc-MAA leak was seen in 33 cases. In the work-up procedure, the following leaks were noticed: 16 into the gallbladder, four into the gastric wall, nine into the duodenum wall, one into the intestinal wall, and three into the abdominal wall (Figure 2). The work-up procedure was repeated in six patients. Five of them have qualified for the radio-embolization procedure (Table 1). 

### 3.1. Gallbladder

**Figure 3:**
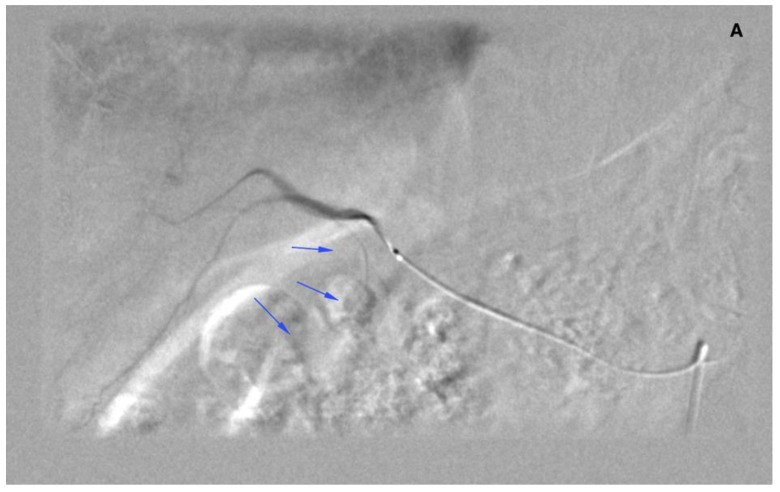

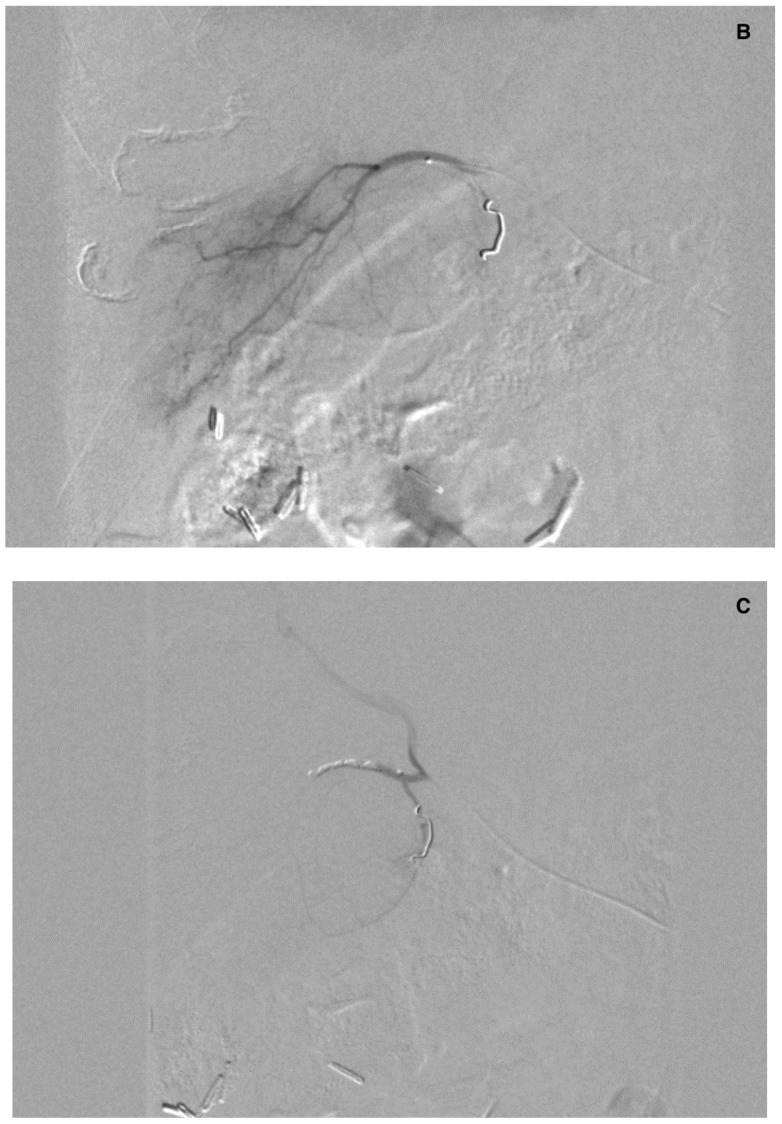

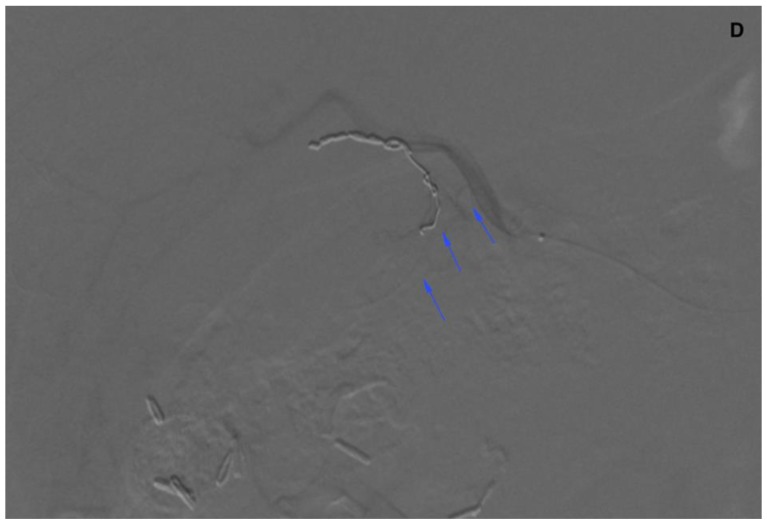
Angiogram of the right hepatic artery. (**A**) Initially, single CA is visible (arrows) originating from the right hepatic artery division. (**B**) After embolization, another artery reveals multiple intrahepatic anastomoses to the gallbladder wall, and needs to be embolized. (**C**) There is insufficient embolization of both CA, and (**D**) retraction of the microcatheter reveals another CA originating proximally to the right hepatic artery division (arrows).

There were 20 patients after cholecystectomy. There were 30 (40%) patients with additional cystic arteries (Figure 3).

Mean distance of the right hepatic artery division from the cystic artery was 4.7 mm (0–35, 6 mm). The cystic artery originated from the right hepatic artery division in 14 patients.

Comparing the position of the tip of the microcatheter to the CA, there were 34 proximal and 31 distal ^99m^Tc-MAA injections resulting in 12 and 4 leaks, respectively (*p* = 0.039, V test, R = 0.31, RR = 2.5, 95% CI 1.2%–5.4). In 54 patients, after CA embolization, there were 30 proximal and 24 distal of ^99m^Tc-MAA injections.

Overall, the mean distance of the tip of the microcatheter from the origin of the cystic artery was 20 mm (minimum of 2.1 mm and maximum of 53 mm) proximally and 10 mm (minimum of 1 mm and maximum of 51 mm) distally (ns); In a subgroup with a leak to the gallbladder, the mean distance of the tip of the microcatheter from the origin of the cystic artery was 14 mm (minimum of 3 mm and maximum of 35.6 mm) proximally and 7 mm (minimum of 2 mm and maximum of 18 mm) distally (ns, compared to the microcatheter position in the subgroup with no leak).

In patients with patent CA, the mean distance of the tip of the microcatheter from its origin was 16 mm (minimum 1.6 mm and maximum 51 mm) distally.

**Figure 4:**
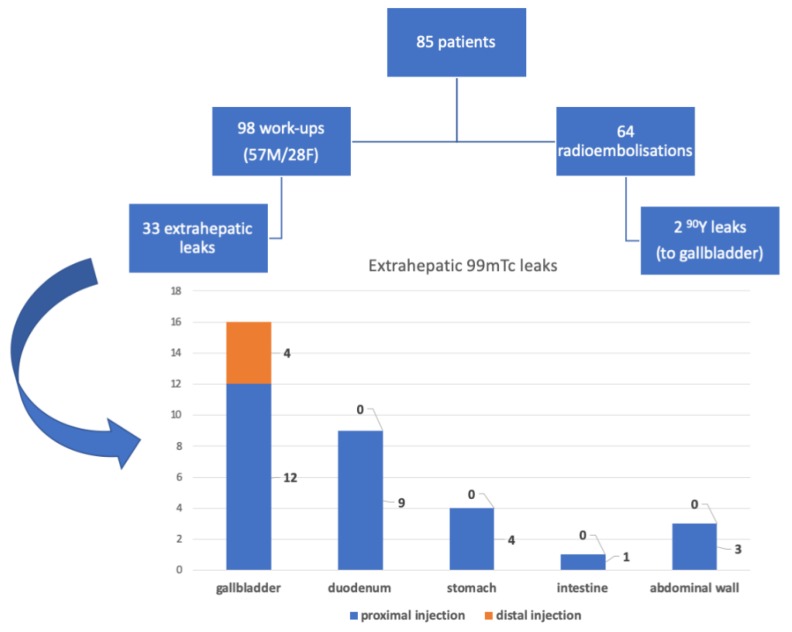
Flowchart presenting the sites of extrahepatic leaks in view of the site of injection.

There were 16 leaks of ^99m^Tc-MAA to the gallbladder wall (15 patients had embolized cystic artery/arteries) *p* = 0.48 Fisher Exact Test. There were no differences in the frequency of gastrointestinal leaks between patients after or without cholecystectomy (gastric-*p* = 0.42, duodenal-*p* = 0.45, intestinal-*p* = 0.14). In 11 patients with patent CA, we observed no leak in two cases of proximal ^99m^Tc-MAA injection, and in one out of nine cases of distal injection (ns). Leak in ^99m^Tc-MAA was also related to the presence of additional arteries (*p* = 0.041, Fisher exact test). For a detailed flowchart, see Figure 4.

A combination of selected predictors: age, gender, prior liver operation, tumor type, Michels type, time between ^99m^Tc-MAA and SIRT, injection direction, distance form noon-target artery and additional arteries, were not significantly associated with a leak to the gallbladder in a multivariate regression model (*p* = 0.054, *R*^2^ = 0.08).

**Figure 5:**
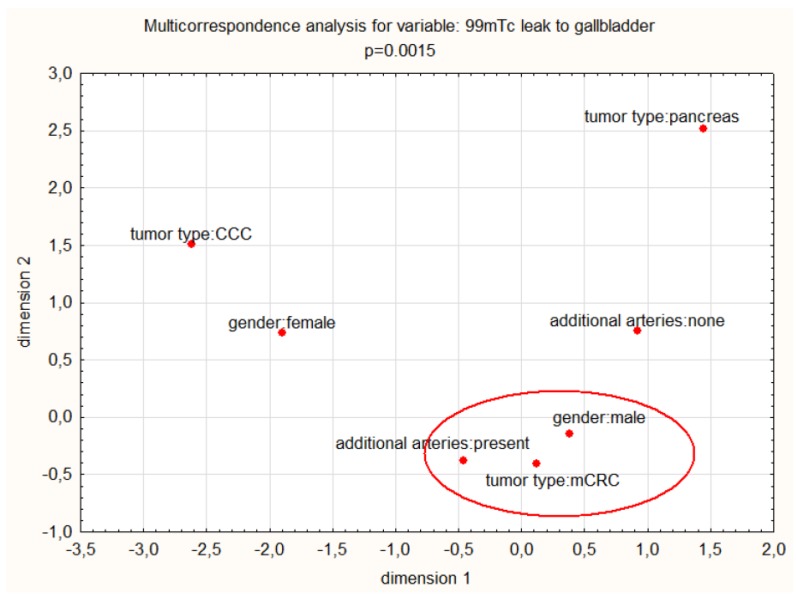
Multiple correspondence analysis for the leak to the gallbladder. mCRC—colorectal cancer metastases. CCC—cholangiocarcinoma.

Nonetheless, additional arteries, male gender, and colorectal cancer metastases have a significant pooled relationship with a CA leak in multiple correspondence analysis (chi^2^ test *p* = 0.00015). See Figure 5.

### 3.2. Bowel Wall

There were 51 RGA and 44 GDA embolizations. The mean distance of the tip of the microcatheter from the origin of RGA was 22.7 mm distally and 11.8 mm proximally, respectively (ns). For GDA, these were 39.5 mm distally and 30 mm proximally, respectively (ns). 

### 3.3. Abdominal Wall

There were three leaks through falciform arteries to the abdominal wall in ^99m^Tc-MAA SPECT/CT. In two cases, dyna-CT confirmed their presence (Figure 5). In one case, there was no sign of contrast enhancement in the abdominal wall. These patients were not given SIRT.

### 3.4. Peri-Procedural Complications

There were three cases of transient pain in the right upper abdominal quadrant, two cases of nausea (both after CA embolization), three cases of hepatic arteries dissection, and seven cases of non-target coil migrations without any clinical symptoms or effect on liver function tests (0–2 CTCAE—Common Terminology Criteria for Adverse Events).

**Figure 6:**
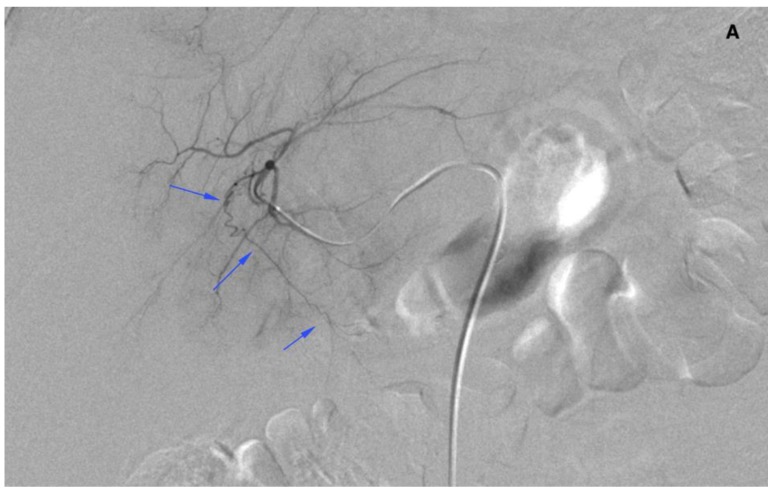

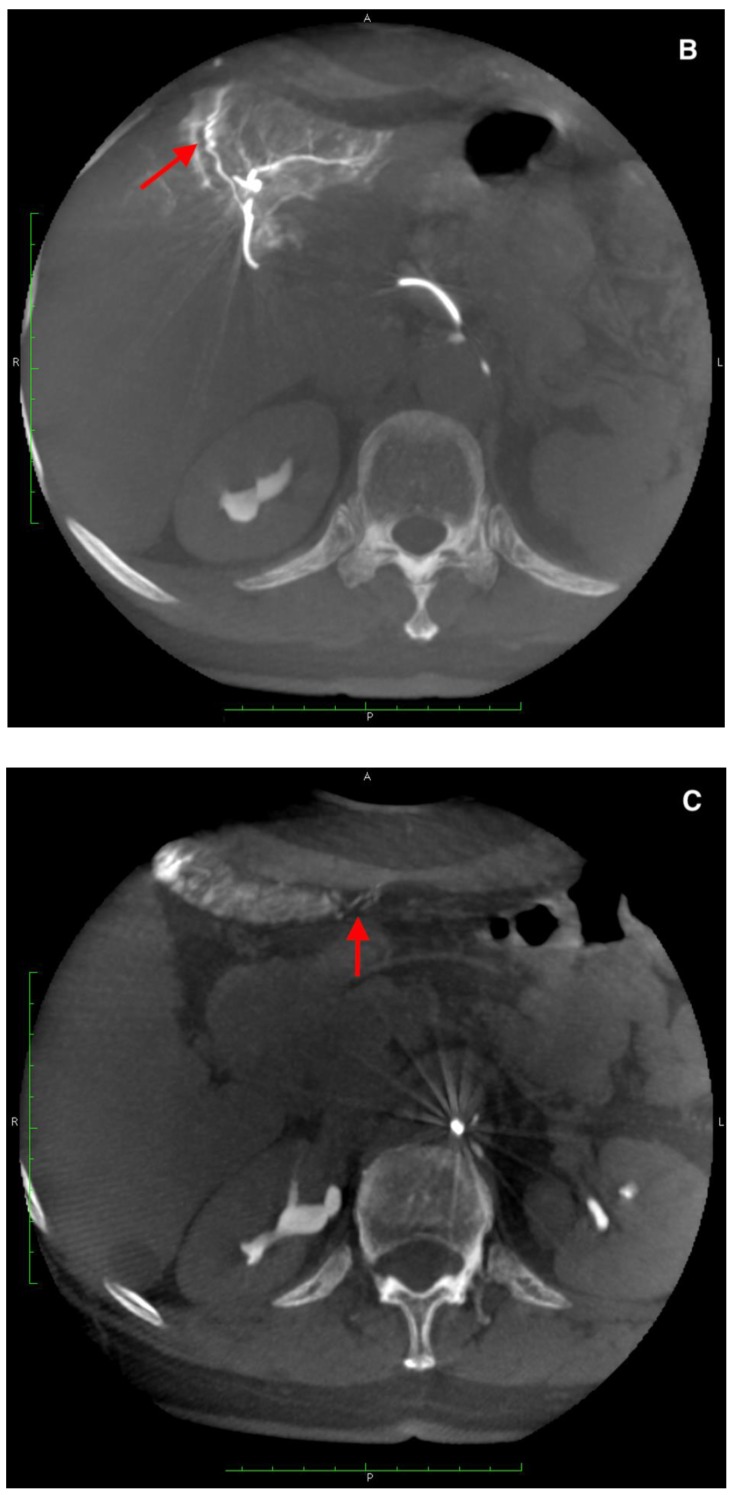

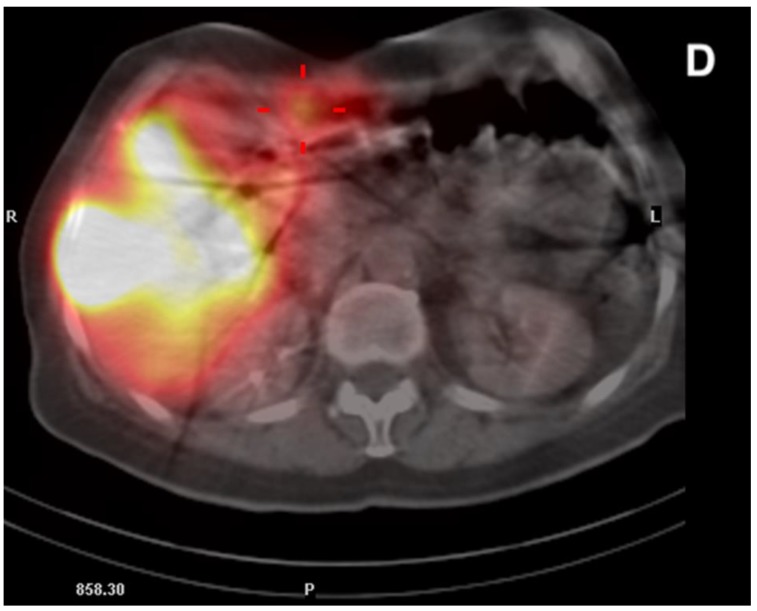
(**A**) Angiogram of the left hepatic artery showing falciform artery travelling medially beyond the margin of the liver. (**B**) Its presence was confirmed on Dyna-CT and showed contrast enhancement of the abdominal wall (arrows) (**C**), and on SPECT/CT after ^99m^Tc-MAA injection (**D**) (*crosshairs*).

**Figure 7:**
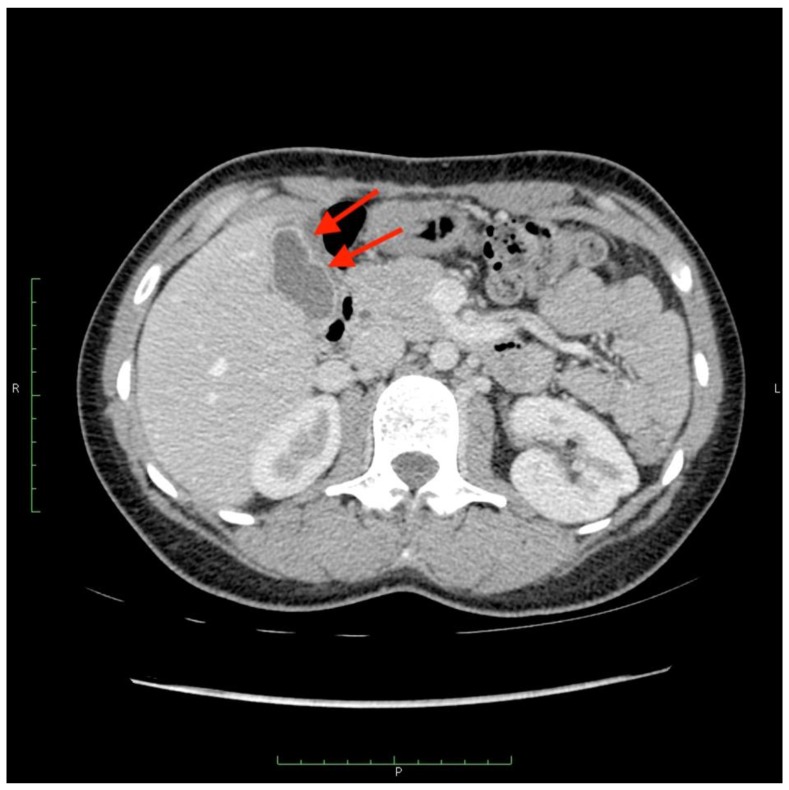
A computed tomography scan depicting asymptomatic cholecystitis (arrows) after the ^90^Y leak. The patient did not require surgery and was treated conservatively.

There were two leaks into the gallbladder wall after SIRT administration. Both patients had no signs of cholecystitis or a bilirubin level elevation (Figure 6 and Figure 7). No leaks were noticed in the group of five requalified patients. 

## 4. Discussion

The goal of this study was to assess quantitatively what constitutes a safe catheter position. The distance of the tip of the microcatheter from coiled or patent non-target arteries was measured during the ^99m^Tc-MAA work-up procedure. 

We noticed 33 extrahepatic leaks. In 16 gallbladder leaks, there were 15 patients after cystic artery embolization. Leaks were strongly related to the position of the tip of catheter (proximal or distal to cystic artery, 12 vs. 4, respectively), regardless of its prior embolization. There was a 2.5 times higher risk of a ^99m^Tc-MAA leak into the gallbladder after proximal injection, with a comparable number of injections (34 vs. 31). In our opinion, CA may prove to be crucial to secure SIRT treatment, if we consider many anatomical variants of its origin. It arises from a distal part of the right hepatic artery in 50% to 75% of cases and is reasonably uncomplicated to find and embolize. In this variant, the tip of the microcatheter may be safely placed distal to the coiled artery to ensure uniform distribution of radiated microspheres within the right hepatic artery branches. Difficulties may occur in case of more distal localization of the cystic artery, especially in division or even in one of the branches of the right hepatic artery [13,15,16]. In this case, ^90^Y-microspheres would have to be injected into two or three branches of the right hepatic artery. It may not only increase the risk of leak of ^90^Y to the cystic wall, but also affects results of the SIRT, because of insufficient ^90^Y deposition within the tumor. In case of several CAs originating from branches of the right hepatic artery, the patient may be even excluded from the treatment.

Paprottka et al. advised to place the microcatheter at least 20 mm distantly from the origin of the non-target artery to secure safe treatment. Yet, there is still a lack of measurable evidence regarding the placement of the microcatheter tip relative to the patent extrahepatic artery [17]. The rule would only apply to GDA, RGA, and, in selected cases, to the supraduodenal artery, where anatomy allows distal placement of the microcatheter.

Our observations do not support the possibility of distal microcatheter placement in case of CA, due to its proximity to right hepatic artery division. We showed that mean distance of right hepatic artery division from the origin of CA was 4.7 mm, i.e., the “20 mm” margin proposed in the literature allows injection only into a selected branch, which is not suitable for uniform distribution of ^90^Y microspheres in the right lobe.

The most difficult scenario in SIRT treatment was the injection of microspheres distal to the patent artery, while their uniform distribution in the vascular bed was maintained. We showed it could be achievable at a minimal distance of 1.6 mm distal from the origin of patent CA, if the injection is thoroughly and patiently monitored for any signs of reflux or changes in flow dynamics. Thus, one could refrain from CA embolization in a seldom case of favorable CA origin anatomy.

This scenario could have been the case in nine patients if CA could not be embolized. In one patient, the microcatheter was placed 18 mm distally to the CA origin, yet there was a leak in ^99m^Tc-MAA. Upon review, it was attributed to additional (not visible initially) CA distal to the microcatheter position. The additional CA was embolized and the patient received SIRT. In other patients, a leak in ^99m^Tc-MAA-SPECT/CT was caused either by the presence of additional artery/arteries missed during the work-up procedure or present collaterals from liver interstitial arteries. 

In the SIRT procedure, there was an inadvertent deposition of ^90^Y-microspheres in the cystic wall in two cases (asymptomatic and handled conservatively). Retrospective analysis of these cases revealed overlooked additional CA arising from the artery supplying segment 6 in the first patient. In the second case, a few tiny feeders arising from the artery to segment 4 were observed. It is worth mentioning that, in this patient, a coil and PVA 100 μm (polyvinyl alcohol) embolization of small arteries in segment 4 was performed during the work-up procedure. Yet, it did not secure him against the ^90^Y-microspheres leak.

Embolization can alter the hemodynamic status of present collaterals, which were not visible at the time of the ^99m^Tc-MAA procedure. Changes in blood flow follow and allow the recruitment of invisible but present intra-hepatic and extrahepatic collaterals. If the time between preparatory and SIRT procedures is long enough, hemodynamic changes following embolization could lead to neoangiogenesis. Newly formed vessels can contribute to extrahepatic leaks in the SIRT procedure [2,9].

Reducing the time gap between work-up and SIRT procedures may potentially lower the chance of opening anastomoses (not necessarily visible on DSA) or forming new vessels, which is ultimately responsible for the leak in SIRT. The mean time between both procedures was 15 days in our group. There are reports in literature, as is our experience, on recanalization of previously coiled arteries, that we observe during the SIRT procedure, which adds to a possible route of an extrahepatic leak. The recanalization rate ranges from 11% to 44% [2]. 

The work-up procedure gives us only suggestions on the microcatheter tip position before SIRT treatment. Safety of the same position of the microcatheter in both procedures should be rechecked and reconsidered. Interventional radiologist should remain cautious during radioembolization and beware of any signs of change in the blood flow in hepatic arteries to avoid blood stasis. In our group of patients, we did not notice any ^90^Y-microspheres leak caused by retrograde blood flow.

Complications arising during or after non-target arteries coil embolization are rare and still not sufficiently described in literature [16]. We observed three cases of pain in the right upper abdominal quadrant, two cases of nausea (both after CA embolization), three cases of hepatic arteries dissection, and seven cases of non-target coil migrations without any clinical symptoms or affection of liver function tests. Adverse events after the SIRT procedure are better described, which include cholecystitis (0.6%–8%), gastrointestinal ulceration (0.7%–28%), or ^90^Y uptake in the anterior abdominal wall (6%—Ahmadzadehfar et al.) [2,17,18,19].

During the first five years of performing SIRT in our center, a rigorous embolization of GDA, RGA, and CA was performed. We resigned from routine coiling of GDA in the first place in our practice. In the vast majority of cases, the origin of GDA was considerably proximal to the microcatheter placement, with a mean distance from the right and left injection position of 26.5 mm and 24.7 mm, respectively. RGA was coiled in most cases, if it was technically possible. Otherwise, a safe distance between the tip of the microcatheter from its origin was maintained. With a mean distance of 22.7 mm (distal placement), we achieved uniform distribution in the left lobe in case of patent RGA. Our observations confirm those made by other authors. These arteries could be safely left patented. With increasing experience, we decided to embolize only the CA when it was localized in the right hepatic artery division or farther. 

Our study has several limitations. At first, it is a retrospective single center study assessing vascular anatomy in a non-uniform group of patients—representing both hyper-vascular and hypovascular tumors. We did not consider the effects of blood pressure, blood viscosity, and heart rhythm on flow dynamics. No sufficient quality of fusion of SPECT/CT images caused by patients’ movements during the procedure may lead to misinterpretations, especially in border areas between the gallbladder and liver tissue (as a shift of SPECT images from the liver tissue over the gallbladder), which may be classified as a lack of ^99^Tc-MAA leakage in some cases. 

Despite limitations, this is the first study to quantitively assess microcatheter placement in patient undergoing ^90^Y-radioembolisation. The study group consisted of a considerable number of patients, which were thoroughly assessed throughout the learning curve of a currently expert-level center.

## 5. Conclusions

A leak in ^99m^Tc-MAA was related to the presence of additional arteries. The microcatheter position within 39.5 and 30 mm from GDA, 22.7 and 11.8 mm from RGA, and 20 and 10 mm from CA allowed safe deposition of ^90^Y microspheres. Special considerations should be taken when non-target arteries remain patent. Regardless of CA embolization, it is 2.5 safer to inject microspheres distal to its origin, compared to proximal injection. The CA origin relative to the right hepatic artery division usually necessitates embolization of the former.

## Figures and Tables

**Table 1:** Data summary for patient’s demographics, anatomy, tumor type, extrahepatic leaks, and measured distances.

Variable	Value (85 Patients)
**Gender (M/F)**	57/28
**AGE**	
mean	58
range	18–78
median	60
**Liver Surgery**	18
**Cholecystectomy**	20
**Tumor Type**	
mCRC	59
HCC	10
CCC	3
melanoma	5
pancreatic adenocarcinoma	3
**Michels Type**	
1	60 (71%)
2	5 (6%)
3	11 (13%)
4	2 (2%)
5	3 (3%)
other	4 (5%)
**Leak in ^99m^Tc-MAA**	
gallbladder	16
stomach	4
duodenum	9
intestine	1
abdominal wall	3
**Leak in ^90^Y Bremsstrahlung**	
gallbladder	2
**Gallbladder**	
CA embolization	54/65 (83%)
Distal position (number/mean, range) (mm)	31/10 (1–51)
Proximal position (number/mean, range) (mm)	34/20 (2,1–53)
**RIGHT GASTRIC ARTERY**	
RGA embolization	51/85 (60%)
RGA Distal position (mean, range) (mm)	22.7
RGA Proximal position (mean, range) (mm)	11.8
**GASTRODUODENAL ARTERY**	
GDA embolization	44 (52%)
GDA Distal position (mean, range) (mm)	39.5
GDA Proximal position (mean, range) (mm)	30
